# Passerine Birds Breeding under Chronic Noise Experience Reduced Fitness

**DOI:** 10.1371/journal.pone.0039200

**Published:** 2012-07-11

**Authors:** Julia Schroeder, Shinichi Nakagawa, Ian R. Cleasby, Terry Burke

**Affiliations:** 1 Department of Animal and Plant Sciences, University of Sheffield, Sheffield, United Kingdom; 2 Department of Zoology, University of Otago, Dunedin, New Zealand; 3 Evolutionary Genetics, Max Planck Institute for Ornithology, Seewiesen, Germany; University of Jyväskylä, Finland

## Abstract

**Background:**

Fitness in birds has been shown to be negatively associated with anthropogenic noise, but the underlying mechanisms remain obscure. It is however crucial to understand the mechanisms of how urban noise impinges on fitness to obtain a better understanding of the role of chronic noise in urban ecology. Here, we examine three hypotheses on how noise might reduce reproductive output in passerine birds: (H1) by impairing mate choice, (H2) by reducing territory quality and (H3) by impeding chick development.

**Methodology/Principal Findings:**

We used long-term data from an island population of house sparrows, *Passer domesticus*, in which we can precisely estimate fitness. We found that nests in an area affected by the noise from large generators produced fewer young, of lower body mass, and fewer recruits, even when we corrected statistically for parental genetic quality using a cross-fostering set-up, supporting H3. Also, individual females provided their young with food less often when they bred in the noisy area compared to breeding attempts by the same females elsewhere. Furthermore, we show that females reacted flexibly to increased noise levels by adjusting their provisioning rate in the short term, which suggests that noise may be a causal factor that reduces reproductive output. We rejected H1 and H2 because nestbox occupancy, parental body mass, age and reproductive investment did not differ significantly between noisy and quiet areas.

**Conclusions/Significance:**

Our results suggest a previously undescribed mechanism to explain how environmental noise can reduce fitness in passerine birds: by acoustically masking parent–offspring communication. More importantly, using a cross-fostering set-up, our results demonstrate that birds breeding in a noisy environment experience significant fitness costs. Chronic noise is omnipresent around human habitation and may produces similar fitness consequences in a wide range of urban bird species.

## Introduction

Anthropogenic noise can acoustically mask, and decrease, the efficacy of avian vocal communication. Warning calls, territorial defence and mating signals can be impaired, and this effect is often indicated by behavioural changes [1]–[6]. Communication impairment can have serious demographic consequences, as it has been shown to result in changes in bird abundance, community structure and predator–prey relationships [7]–[9]. More importantly, noise can also affect reproductive output. In a population of great tits (Parus major), for example, females laid smaller clutches in areas affected by traffic noise than in quieter areas; also, nests in noisy areas fledged fewer young [10]. The underlying mechanisms, however, remain unclear (but see [11]). Thus, while it is interesting to consider the effects of noise on specific behaviours, it is crucial to conservation efforts in urban environments to study the direct effects of environmental noise on reproductive success and recruitment [12].

Three, non-mutually exclusive, hypotheses have been suggested to explain why reproductive success is reduced in noisy areas [10]. H1, *impaired mate choice hypothesis*: Noise may interfere with the transmission of mate quality through bird song and a female’s assessment of the quality of her mating partner may be impaired [10], [11]. Under this hypothesis, females are expected to invest less, lay smaller clutches and solicit more extra-pair copulations when breeding in a noisy environment. H2, *reduced territory-quality hypothesis*: Noise may affect territory quality. If this is true, noisy areas are expected to be populated by less experienced or younger individuals of lower quality, or to be avoided in general [8], [12], [13]. H3, *impaired chick development hypothesis*: Noise can lead to poor chick development, by means of two different pathways. First, noise can induce physiological stress in chicks, which may lead to reduced growth [14]. Second, noise may mask acoustic communication between offspring and parents. Two potential mechanisms can operate: if chick begging is not audible, or is less audible, because it is acoustically masked by background noise, we expect chicks to increase the amplitude of their begging, or parents to provision less frequently [10]. Another possibility is that chicks may fail to notice their parents’ arrival at the nestbox, resulting in them not begging for food [15].

These three hypotheses each predict reduced reproductive success in noisy environments. Here, we test these three hypotheses in an altricial passerine, the house sparrow (*Passer domesticus*). It is not usually possible to test for a within-individual effect of a noisy environment in a wild population, because this would usually require either the relocation of breeding individuals from a quiet to a noisy environment, and vice versa, or the experimental modification of the noise level around a group of breeding individuals [11]. The relocation of breeding birds is generally impractical. Changing the background noise level via loudspeakers would make it difficult to distinguish between the effects of the noise treatment *per se* and the effect of disturbance due to a change in the noise environment. Here, we take a different approach: we have a dataset of repeated measurements on individual sparrows who have bred in a noisy and three quiet environments, which, together with a cross-fostering set-up, allows us to statistically distinguish between among- and within-individual effects, as well as separating the effects of individual genetic quality and environmental noise. These data allow us to study the direct reactions of birds to the environmental noise that is part of their normal environment.

## Methods

We used data from a long-term (2001–2008) study on a nestbox population of house sparrows on Lundy Island [16]–[21]. Low levels of migration to and from the island allow for accurate fitness and recruitment estimates; annual resighting probabilities of marked individuals are extraordinarily high (average 0.91, range: 0.72–1.00, [21]). The population has been systematically monitored since 2000; all nesting attempts are recorded from the moment the first egg is laid. Nearly all birds are individually marked as fledglings – therefore, we know their exact ages [17]. Cross-fostering of 2-day old hatchlings between nests has been routinely carried out between randomly chosen clutches of the same age, without changing clutch size, since 2000. Cross-fostering is a routine and systematic component of Lundy sparrow fieldwork and was not restricted to specific experiments (for more details on two small experiments please refer to [21] and references within). Birds were considered to have recruited into the breeding population if they started a brood.

**Figure 1:**
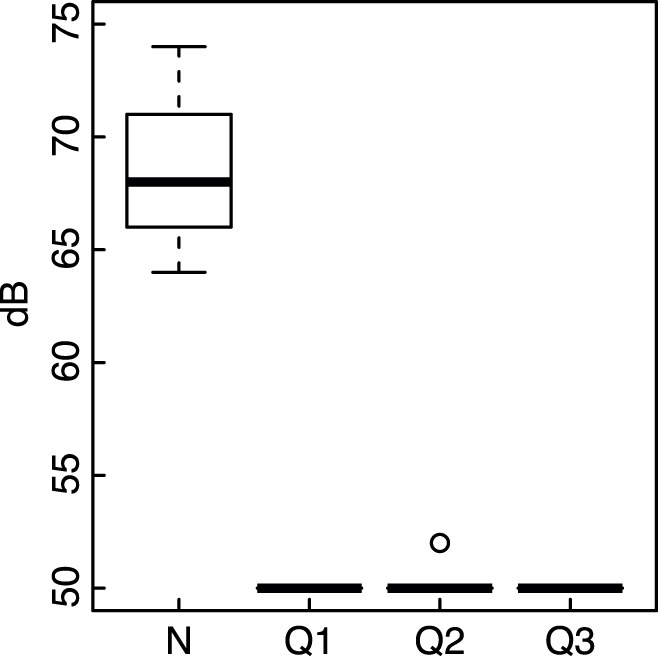
Mean noise levels at the four sites. Noise levels were assessed during the breeding season and measured at five random nestboxes at each site. We used a Silverline sound level meter, with A-weighting, with a range from 50–126 dB and an accuracy of ±2 dB.

**Figure 2:**
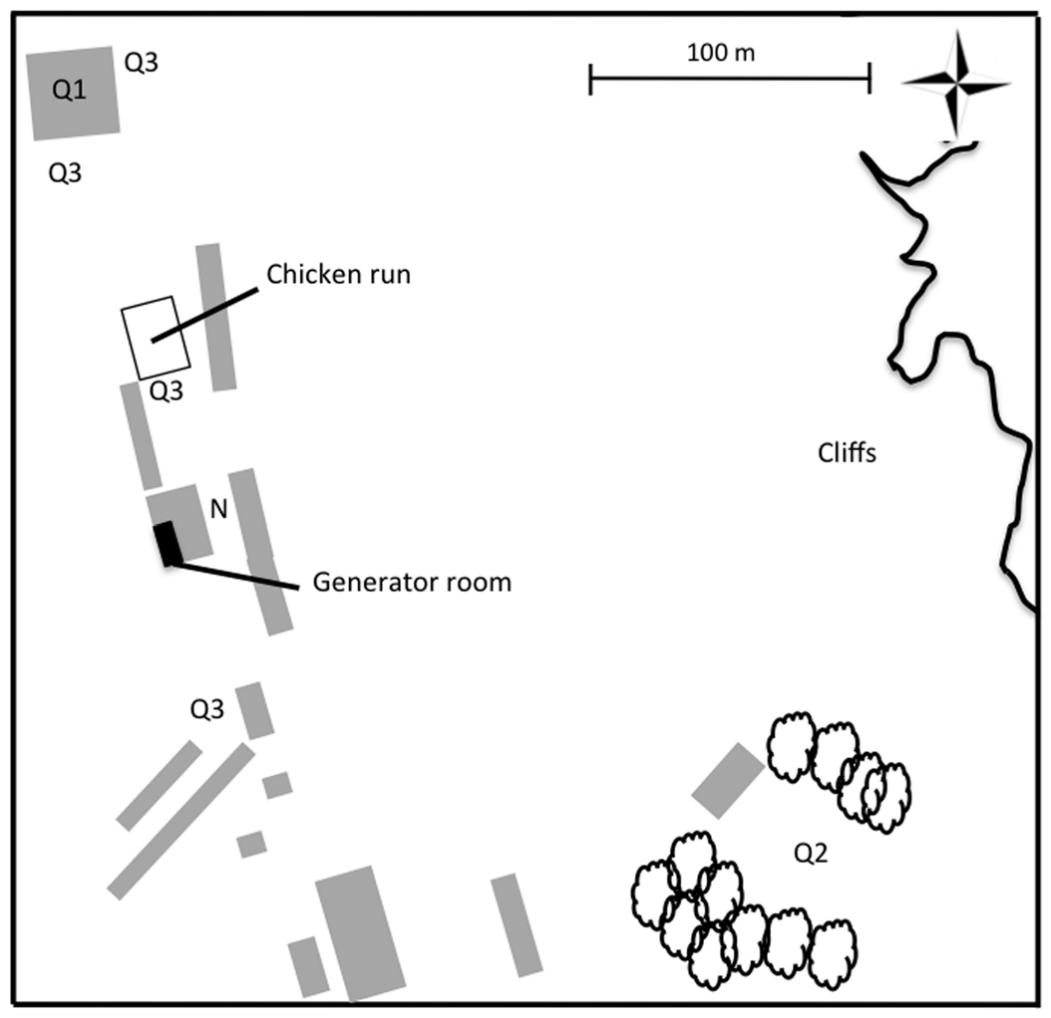
Locations of house sparrow nestboxes on Lundy Island. Grey boxes depict buildings.

Lundy Island is not connected to the power grid and electricity is generated on the island. Since March 2001, a set of generators (Cummins 6DTA5.9 and 6CTA8.3) has been run continuously between 06∶00–12∶00 h each day. These generators produce low-frequency noise that reverberates in the adjacent area (noisy environment, N), producing on average 68 dB(A) at the entrances of 29 nestboxes in the barn (Figs. 1, 2), as measured with a hand-held Silverline sound level meter. Another barn (quiet, Q1) harbours 46 boxes; 28 other nestboxes are attached to the outside of the buildings (Q2) and a further 27 nestboxes are located in a small wood (Q3). In Q1–Q3 the generator is only slightly audible. All areas but Q3 are similarly close to the main foraging area, the chicken run (Fig. 2).

The identities of parents at nestboxes were determined by visual identification of individual colour-ring combinations (viewed directly or with the help of video recordings), by catching parents at the nest box [17], and by using PIT-tags and corresponding nest-box antennae [21]. Since not all parents were caught at nestboxes the sample sizes for morphological measurements of parents differed from the sample sizes for parents of known age. Provisioning and incubation frequencies (measured as visits per hour), and incubation duration (in minutes) have been quantified since 2004 from video recordings (90 minutes long) taken at the nestboxes. The methodology is described in detail in [16]. Since sparrows are multi-brooded and, once in the breeding population, live on average for 3–4 years [17], we have repeated measures of provisioning by the same individuals within and between years, which allows us to test whether the same individuals changed their behaviour when they bred in the noisy area *vs* the quiet area. For the main analysis, we used provisioning frequencies collected at broods containing chicks that were 7 days old. We used Bayesian Markov-chain Monte Carlo (MCMC) methods to fit mixed models (BMM). We report effect sizes of the means of the posterior distribution. We considered fixed effects to be statistically significant if their 95% credibility interval (CI) did not include zero [22]. We used R 2.12.1 for statistical analyses.

### Fitness Consequences of Noise

We first tested for the fitness consequences of being reared in a noisy location, independent of any potential mechanism. We only used cross-fostered chicks in this analysis. We compared the fate of chicks reared in the noisy environment, N (coded as 1), with those of birds breeding elsewhere, Q (all quiet areas pooled, coded as 0 =  reference level). We used two binomial BMMs, with respectively survival from nestling to post-fledging and recruitment as the binomial response variables (survived  = 1) and foster area (noisy versus quiet) as a fixed factor. We modelled year and natal area as random effects to correct for potential differences in parent quality. We modelled natal brood as a random effect to correct for chicks from the same nest being more alike than those from different nests.

### Reproductive Investment (H1)

We tested if females invested differently in reproduction depending on whether or not they bred in the noisy environment. We tested for a difference in incubation visits and incubation time, whether broods in noisy areas contained fewer eggs and hatchlings, and whether the seasonal timing of breeding differed. We used data on genetic parentage [17] to test whether females breeding in the noisy area had more extra-pair offspring than those breeding in other areas.

### Territory Quality (H2)

We tested whether sparrows avoided breeding in the noisy area by comparing annual occupancy rates between the areas. We then examined for the possibility that low-quality or less-experienced birds bred in noisy areas by comparing body mass and the age of parent birds breeding in different areas.

### Chick Development (H3)

We first tested for the expectation that chicks that experienced noise grew more slowly, and tested for differences in body mass between fledglings from the noisy areas and elsewhere. We used only chicks that had been cross-fostered. We used a Gaussian BMM with brood, natal area and cohort as random effects to assess the effect of noise on chick body mass at day 12 after hatching. We corrected for time of day (morning or afternoon) as a fixed effect because chicks were lighter at the start of the morning before their parents started provisioning.

We then tested whether parents provided less to broods in a noisy environment than elsewhere. We carried out a cross-sectional analysis with Gaussian BMMs, where we compared the provisioning frequencies of sparrows breeding in the noisy environment with those breeding elsewhere in two models, one for each sex. We corrected for age of the parent and day of season by adding both variables as fixed effects to the model. Bird identity was modelled as a random effect on the intercept, as was year, to correct for annual variability. We then added identity of the partner as a random effect on the intercept, to correct for a potential bias resulting from the adjustments that individuals make, depending on the degree of parental investment by their partner [23].

### Within-individual Effects of Noise on Provisioning

Using the same data, in which we have multiple records of individuals, we compared the provisioning by individual parents with those by the same individuals breeding in different areas (within-individual effects), using within-subject centring of variables in BMMs [24]. This model tests for the possibility that individual birds may display high provisioning frequencies when breeding in a quiet area, but low provisioning frequencies when breeding in a noisy area (either in the same or in subsequent years). We modelled the provisioning frequency of males and, respectively, females, as response variables. Our basic model structure was similar to the cross-sectional model, but did not include the non-significant effects of age, date of season, and identity of the partner. We added the number of chicks as a covariate, as individual birds may be able to flexibly adjust their provisioning frequency depending on the number of chicks they feed. We modelled bird identity as a random factor on the intercept, to account for potential heterogeneity among individuals. We used two new variables as fixed predictors: to eliminate any between-subject variation, we subtracted the mean location value (coded as: noisy  = 1, quiet  = 0) for each individual across all its broods from the value for the location of each individual brood. That is, if a female bred once in the noisy environment and once elsewhere, it would get the value 0.5 for the datum when breeding in the noisy environment, and −0.5 for the other datum. This term estimates the within-subject variation component. We derived a second predictor variable to estimate the between-subject variation in provisioning, which is the mean area code for one individual [24].

To test whether within- and between-individual effects differed, we used a similar model, modelling the location (noisy or not) of each brood as a within-individual term and the mean location term from the first model, which represented the difference between the within- and between-individual effects. In both within-individual models, we also corrected for the number of hatchlings.

In order to test whether noise is the causal agent for the reduction of provisioning rate, we re-analysed the video recordings of two nests affected by the intermittent noise produced by a set of large industrial ventilators responsible for sucking in air to cool the power generators. When present, the noise level experienced at these separate nestboxes averaged 70 dB(A). The fans are turned on and off automatically as needed, at times of increased power consumption. Note that the nests are not affected by any airflow from these ventilators. We identified 22 video recordings of these nestboxes in which the ventilators either switched on or off; this was easily identified by listening to the audio track. The time when the fans went off or on was recorded. We calculated provisioning rate separately for the noisy and quiet sections of the videos, and tested whether birds responded directly to the noise levels. Provisioning frequency and fan use might be linked through a common correlate, such as outside temperature. In order to account for such a possibility, we used other videos taken at the same time, but at quiet nestboxes, as controls. This was possible because we usually used two or more cameras, and, therefore, matching videos were available for most cases. We partitioned the time in the same way as we partitioned the video data at the noisy locations. We then tested in the controls for a change in provisioning frequency during the times when the fans were on, even though those nests were not afflicted by the noise. For this analysis, we used data on provisioning frequencies across all chick ages to increase sample size.

This work was carried out under the permit from Natural England 20092529.

## Results

### Fitness Consequences of Noise

**Figure 3:**
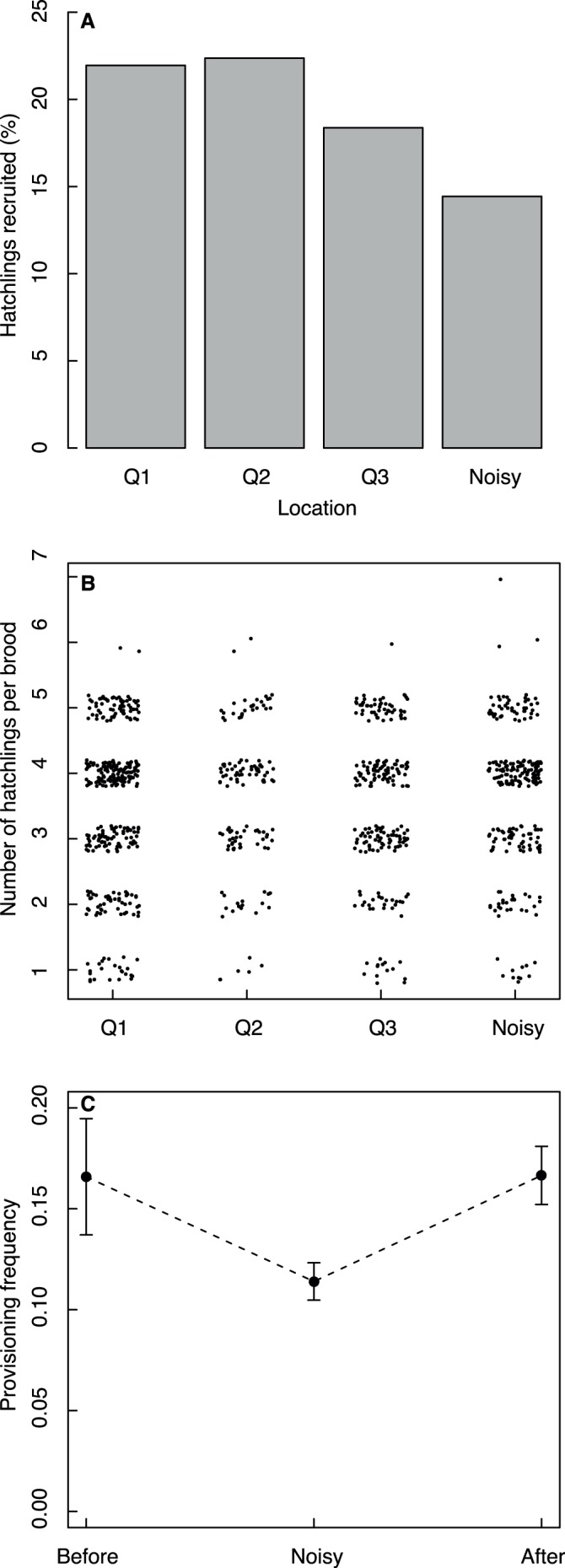
Reproductive success and provisioning frequency of Lundy island house sparrows breeding in nestboxes in the noisy area and elsewhere. (a) Percentage of house sparrow hatchlings that recruited to the breeding population, in relation to the environment in which they were raised (Q1–3 =  quiet, N  =  noisy). These data are not corrected for natal brood and foster area. (b) Number of Lundy house sparrow hatchlings per brood in relation to brood area (jittered). (c) Provisioning frequency (visits per minute) within individual female house sparrows that bred in quiet environments before and after they bred, or both, in the noisy environment. *N*  = 69 females switched between noisy and non-noisy locations between broods. Whiskers depict one standard error.

We compared the fate of cross-fostered house sparrow chicks reared in a noisy environment with those reared in other places (Figs 1, 2). Being reared in a noisy environment was associated with a significant drop in survival between hatching and fledging: When correcting for natal brood and area, the probability of fledging was 0.25 for nestlings reared in quiet environments (*N*  = 1093) and 0.21 for chicks reared in the noisy environment (*N*  = 381, Table 1, back-transformed coefficients from a binomial mixed linear model [23]). Chicks reared in the noisy environment also had a statistically significantly lower probability of recruiting into the population, compared to chicks from the other areas (Table 1, Fig. 3a).

**Table 1:** Results of a BMM with a logit link function modelling fledging and recruitment probability, of cross-fostered Lundy Island house sparrow chicks as response to noisy and quiet environments.

	Fledged		Recruited	
Effects	Posterior mode	95% CI	Posterior mode	95% CI
Fixed				
Intercept	1.02	0.48 – 1.39	−1.73	−2.30 – −1.08
Noisy environment	−0.55	−0.94 – −0.17	−0.49	−0.78 – −0.22
Random				
Brood	3.2	2.48–4.14	0.94	0.51–1.34
Natal location	0.01	0.00–0.12	0	0.00–0.02
Cohort	0.22	0.01–1.12	0.29	0.12–1.65
Residual	0	0.00–0.17	0.02	0.01–0.06

The quiet environment is the reference level. Statistically significant fixed effects are indicated in bold. *N*  = 1474 chicks.

### Reproductive Investment (H1)

Broods in the noisy area did not differ from broods in quiet areas in the number of eggs (ANOVA with area (N, Q1–3) as a factor: *F*_3,1052_ = 0.24, *P*  = 0.87), the number of hatchlings (*F*_3,967_ = 1.12, *P*  = 0.34, Fig. 3b), or the laying date (*F*_3,1135_ = 1.13, *P*  = 0.34). The number of incubation visits did not differ between noisy and quiet environments (Kruskal-Wallis test, Males: *χ* ^2^ = 1.13, *df*  = 1, *P*  = 0.29, *N*  = 66; Females: *χ* ^2^ = 2.06, *df*  = 1, *P*  = 0.15, *N*  = 66). Also, male and female house sparrows spent a similar amount of time incubating broods in the noisy environment as elsewhere (Males: *F*_1,65_ = 0.02, *P*  = 0.89; Females: *F*_1,65_ = 0.40, *P*  = 0.53). The proportion of clutches that contained extra-pair eggs did not differ between the noisy and the quiet environments (estimates from a binomial BMM with noisy or not as a fixed factor and year as a random effect: fixed effect: *b* *_intercept_*  = −1.27 (−5.46 to −0.45); *b* *_noisy_*  =  −0.54 (−1.86 to 0.80), *u* *_year_*  = 0.62 (0.16 to 19.47), *e* *_residual_*  = 0.22 (0.08 to 39.12), *N*  = 953 broods in 10 years). Also, the number of eggs per clutch sired by a male other than the social father did not differ among the four areas (Poisson BBM, fixed effect: *b* *_intercept_*  = 0.10 (0.05 to 0.16); *b* *_noisy_*  = 0.002 (−0.02 to 0.04), *u* *_year_*  = 0.35 (0.07 to 3.60), *e* *_residual_*  = 0.48 (0.33 to 0.79), *N*  = 953 broods).

### Territory Quality (H2)

Annual occupancy rates of nestboxes did not differ between the noisy area and elsewhere (ANOVA *F*_3,36_ = 1.09, *P*  = 0.37). Body mass of sparrow parents was similar between quiet and noisy areas (females: *F*_3,584_ = 0.15, *P*  = 0.93; males: *F*_3,520_ = 0.98, *P*  = 0.40). Female age did not differ between noisy and quiet areas (Kruskal-Wallis test: *χ* ^2^ = 0.32, *df*  = 1, *P*  = 0.57, *N*  = 962) but males breeding at the noisy areas were older than those breeding elsewhere (Kruskal-Wallis test: *χ* ^2^ = 7.09, *df*  = 1, *P*  = 0.01, *N*  = 954).

### Chick Development (H3)

We compared the fledging body mass of chicks reared in a noisy area with those reared elsewhere. We used the data from our cross-fostering experiment and corrected for the location of the natal brood. This was done to distinguish between the effect of low-quality parents, which might produce low-quality offspring, breeding more often in the noisy environment than elsewhere, from chicks suffering from being reared in the noisy environment. Chicks that were reared, but not necessarily born, in a noisy area had a significantly lower body mass when 12 days old than chicks reared in a quiet area (BMM, body mass at day 12 in grams: fixed effects: *b* *_intercept_*  = 23.91 (23.12 to 24.80); *b* *_noisy_*  =  −0.74 (−1.39 to −0.02), *b* *_time of day_*  = 1.58 (0.77 to 2.24); random effects: *u* *_brood_*  = 5.52 (4.77 to 7.73), *u* *_natal area_*  = 0.01 (0 to 0.02), *u* *_year_*  = 0.005 (0 to 0.98), *e* *_residual_*  = 7.49 (6.82 to 8.55), *N*  = 922).

#### Cross-sectional analysis

We then compared the provisioning frequencies of house sparrows breeding in the noisy environment with those of birds breeding elsewhere. Females, but not males, provisioned broods in the noisy environment significantly less often than in other areas (Table 2). Consistent with the previous observation that males are more repeatable in their parental care than females [16], we also found that males were individually more predictable caregivers than females (Table 2).

**Table 2:** Results of a BMM modelling Lundy island house sparrow provisioning frequencies (visits/hour) on day 7 in quiet and noisy environments. Statistically significant fixed effects are indicated in bold.

	Female provisioning frequency		Male provisioning frequency	
Effects	Posterior mode	95% CI	Posterior mode	95% CI
Fixed				
Intercept	8.91	5.77–10.63	7.24	4.49–11.03
Noisy environment	−2.31	−3.20– −1.51	−0.85	−2.31–0.27
Laying date	0.009	−0.01–0.02	0.004	−0.01–0.02
Age of Mother	0.23	−0.12–0.56	0.14	−0.21–0.61
Age of Social Father	−0.16	−0.43–0.14	−0.16	−0.50–0.35
Random				
Mother ID	0	0.00–0.74	0.002	0.00–0.75

Females: *N*  = 422, with observations on 147 individuals; males: *N*  = 420, with observations on 138 individuals.

#### Within-individual effects of noise on provisioning

We used the same data to compare provisioning frequencies of individual parents breeding in the noisy area with the provisioning frequencies of the same individuals when they bred elsewhere (within-individual effects, see [24]). Individual females visited their broods less often per hour when breeding in the noisy environment (BMM parameter estimates (CI), fixed effects: *b_intercept_*  = 6.52, (4.93 to 8.01); *b_within_*  =  −1.09 (−1.60 to −0.62); *b_between_*  =  −1.30 (−2.41 to −0.29); *b_clutchsize_*  = 0.40 (0.02 to 0.78), random effects: *u_ID_*  = 0.39 (0 to 1.44), *u_year_*  = 0.07 (0–0.34), *e_residual_*  = 16.61 (14.3 to 19.46)). The within- and between-female effects of breeding in the noisy environment were not significantly different (BMM: fixed effects: *b_intercept_*  = 7.86 (6.10 to 9.56); *b_within_*  =  −2.59 (−4.81 to −0.59); *b_? between-within_*  = 0.21 (−1.09 to 1.35); *b_clutchsize_*  = 0.39 (−0.01 to 0.75). We did not find a similar effect of noisy location on provisioning frequency in male house sparrows (BMM, fixed effects: *b_intercept_*  = 6.82 (4.32 to 9.47); *b_within_*  =  −0.88 (−3.95 to 2.13); *b_between_*  =  −0.10 (−1.82 to 1.80); *b_clutchsize_*  = 0.61 (0.19 to 1.14), random effects: *u_ID_*  = 4.69 (1.84 to 8.20), *u_year_*  = 0.71 (0 to 3.37), *e_residual_*  = 24.13 (20.91 to 28.29)).

We then used a subset of data that consisted only of those females that changed, within and between years, from or to the noisy area, and tested whether their provisioning frequency changed. We retained the information on whether or not females had bred previously in a quiet area and moved into a noisy environment, or *vice versa*. The same females provisioned their young more frequently before and after moving to the noisy environment (*N*  = 96 broods, Figure 3c, linear model with area as factor: *F*_1_ = 11.48, *P*<0.001; clutchsize: *F_1_* = 0.24, *P*  = 0.63).

**Figure 4:**
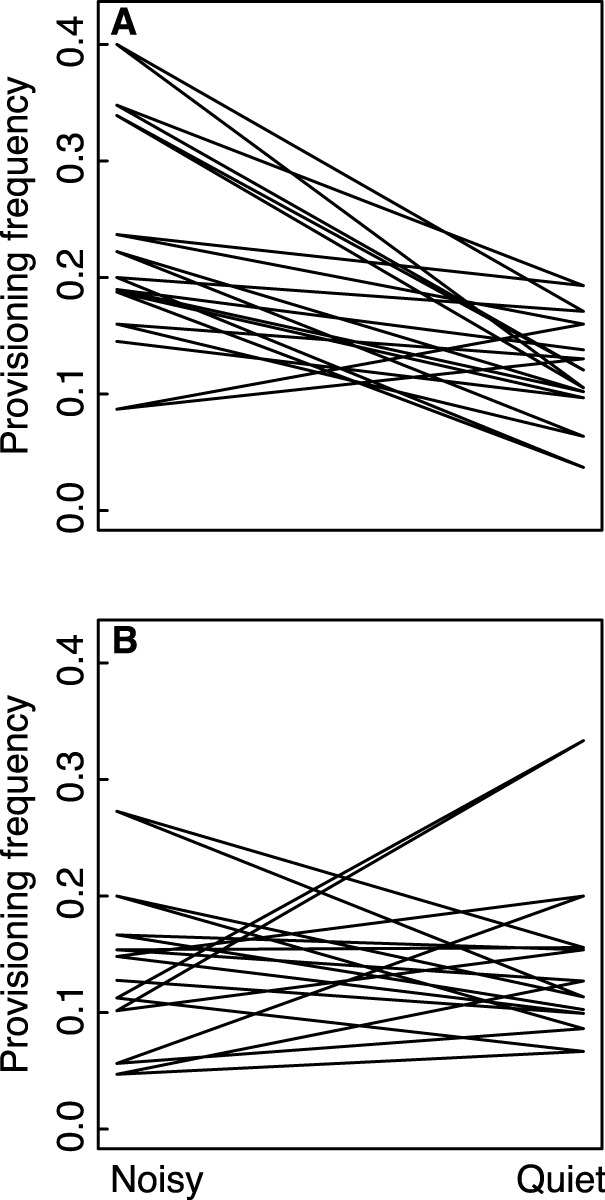
Frequency of provisioning (visits per minute) by female Lundy island house sparrows breeding in nestboxes affected by intermittent noise (top), and by those not affected by noise. Provisioning frequencies were calculated for the time period during which the noise was on and off in both groups. Lines represent changes in provisioning rate within individual females.

Finally, we also tested whether sparrow females reacted by adjusting their provisioning rate in response to short-term noise. When the noise was present, female sparrows had a reduced provisioning rate within a single brood, and an increased provisioning rate when the noise-producing ventilators were off independent of the sequence of events (Fig 4, Table 3). We used data from video recordings of provisioning taken at quiet locations but at approximately the same time as controls, because the fans’ running time might have been correlated with some external variable that also affected provisioning. However, we found no change in provisioning rates at quiet nestboxes during the times when the fans were on (Fig. 4, Table 3).

**Table 3:** Results of a BMM modelling Lundy island house sparrow female provisioning frequencies (visits/hour) at a location intermittently affected by noise and at control nestboxes during the same time periods (two-level factor with noise off as the reference level). Statistically significant fixed effects are indicated in bold.

Effects	Effect size 95% CI	Effect size 95% CI
	Intermittent noise		Control	
Fixed				
Intercept	13.73	10.54–16.60	8.27	5.44–10.90
Noise on	−6.54	−10.48–−2.61	0.25	−3.44–4.52
Random				
Bird ID	1.54	0.00–7.74	0.26	0.00–4.39
Residual	21.92	9.18–37.71	20.63	8.40–35.73

*N*_noise_  = 22 observation periods on five females, N_quiet_  = 20 observation periods on nine females.

## Discussion

House sparrows reared in a noisy environment experienced reduced parental provisioning, lower fledging mass, and lower fledging and recruiting success. Our results support the impaired chick development hypothesis (H3). We observed a reduced provisioning frequency in the noisy environment, which is suggestive evidence for a novel mechanism of how noise may affect fitness of passerines: by masking parent–offspring communication. Our study has one caveat: We had only one location that was subjected to constant noise with sufficient data to measure fitness, and we can therefore not exclude the possibility that some other variable we did not account for caused the drop in fitness in the noisy area. We, however, do not believe that this is the case because the noisy location is similarly close to the main feeding grounds as most other nest sites and, therefore, birds should not have had a harder time foraging. If another environmental factor, such as exhaust pollution, caused the lowered condition of chicks then we would have also expected to see a similar effect in the physical condition of adults breeding in that area, which we did not find. Similarly, if another factor had led to a change in the visitation rate by birds to their nests, we would also have expected a difference in incubation visits between the noisy and quiet areas, which again we did not find. Furthermore, all nestboxes in all areas were built following a standard model [25], reducing environmental variability. We have found earlier that house sparrows on Lundy are consistent in their within-individual reproductive output between years, which indicates that deviations from this constancy may be due to changes in the environment, not changes in the adult [17]. Finally, the observation that females respond flexibly to the presence of noise within short periods of time supports the idea that a change in feeding rate, as a response to noise, might be the cause for the low fitness in the noisy area.

We did not find support for the impaired mate choice hypothesis (H1): females did not decrease reproductive investment other than provisioning behaviour when breeding in the noisy area: clutch size, breeding date and incubation behaviour did not differ between the noisy and quiet areas. Clutches in the noisy area did not contain an increased rate of extra-pair offspring (contradicting [11]). It is possible that females decreased provisioning rate in response to a potentially perceived low mate-quality, if mate quality in house sparrows is mainly signalled by song displays. Little is known about how song quality affects female choice in house sparrows. However, if coitus, and the decision to mate with a certain individual, take place away from the nest [26] and outside of the noisy environment in our study, it is likely that most females have the chance to sample their mate’s song quality in a quiet area, unbiased by the noise. Furthermore, if females assume her mate is of lower quality it would be more prudent to reduce primary reproductive investment, i.e. in clutch size rather than reducing parental care after investing in costly eggs. The similar rates of extra-pair offspring between nests in noisy and quiet areas additionally suggest that mating decisions of females were not affected by the noise. We therefore assume that, in our study, acoustic masking of the communication between the adults probably did not affect the reduced reproductive fitness in the noisy environment.

We found no support for the impaired territory quality hypothesis (H2): Sparrows did not avoid breeding in the noisy area. Surprisingly, we found that older males, but not females, were more likely to breed in the noisy environment. Older house sparrows have a larger black bib, which signals social dominance [18], [27]. The apparent preference of older males for the noisy area is difficult to explain, although it must be noted that the effect size was relatively small (0.3 years difference). However, assuming that older males are of higher quality, they would seem to consider the noisy area to be a desirable habitat.

Our results support the impaired chick development hypothesis (H3). Our study set-up does not allow us to distinguish between the effects of chronic stress and those of acoustic masking, and we discuss supporting evidence for or against both possible mechanisms. Chronic noise is known to induce stress-related changes along the hypothalamo–pituitary–adrenal axis [14], which might influence chick and parent physiology. We found twelve-day-old chicks to be of lower body mass when reared in the noisy environment, however, this seems as likely to be a consequence of the reduced provisioning frequency as a reaction to chronic stress. We found no evidence for an effect of stress in adults: body mass of adults did not differ between the noisy and quiet areas, which indicates that, at least for adults, the noise did not result in lowered condition due to stress. Stress could also affect adult behaviour and nest visitation rates. If this were the case, we would expect this stress response to similarly affect incubation behaviour, which was not the case. We cannot exclude that chronic noise and the associated stress has been the sole cause for the lowered chick condition, but given our results we consider it unlikely.

Provisioning rates were lower in the noisy environment than elsewhere. We have also shown that sparrow females respond flexibly to short-term, familiar environmental noise with an immediate reduction in provisioning frequency. The observation that sparrow females increase their provisioning rate during times with no noise is suggestive evidence for a causal mechanism to link provisioning behaviour with environmental noise. Parental birds use the information communicated to them through begging from their chicks to adjust their provisioning frequency according to the chick’s needs [28]–[33]. Offspring begging is an adaptive behaviour [33]; parent birds increase their provisioning rate when presented with increased begging [30]. Therefore, if noise masks begging vocalisations, parents will not respond appropriately. Another possibility is that offspring may not hear their parents arriving at the nestbox and therefore fail to beg for food [15].

We only found females to lower their provisioning rate in the noisy environment, not males. In house sparrows, males provide food to their young at a relatively constant rate while females are more flexible [16]. The most parsimonious explanation for the differences between the sexes is that males are unresponsive, while females may be more responsive to nuances in the chicks’ begging vocalisations. We suggest that, in the noisy area on Lundy, female sparrows perceive they have less needy chicks because the acoustic communication with their chicks is intercepted by generator noise. The chicks of unresponsive parents are disadvantaged [29], [32], [33]. We suggest that acoustic masking of parent–offspring acoustic communication may be at least a partial explanation for the lowered parental provisioning in the noisy areas.

The strength of our study is that it suggests direct fitness consequences of chronic noise in wild birds. Fitness is generally difficult to measure in wild populations but, by using an island population, we can be relatively sure that the birds affected by noise had not simply dispersed. It is perhaps surprising that such a large fitness effect is found in house sparrows, a species thought to be well adapted to living in close association with humans, where chronic background noise is pervasive. Yet, insufficient reproductive output has been shown to be responsible for the decline of the sparrow from cities and rural areas [34]. Factors associated with urbanisation and food availability have been suggested as causes [35]. Our results point to the possibility that chronic noise might be a part of the explanation for the decline of the house sparrow in urban areas. Urban noise has been shown to interfere with acoustic communication between conspecifics in several bird species [9], [36]. In order to assess which particular urban noises could be problematic we would need a comprehensive acoustic analysis of sound frequencies. The potential of urban noise to acoustically mask parent–offspring communication, as well as the physiological effects of urban noise, need to be investigated experimentally in order to validate the extent of these effects, and to understand the conservation implications [12].
